# SELENOP-LRP8-mediated macrophage-endothelial cell interaction promotes breast cancer vascular invasion by inducing metabolic reprogramming in endothelial cells

**DOI:** 10.1007/s00018-026-06359-y

**Published:** 2026-07-31

**Authors:** Haoran Wang, Yuan Song, Mengdi Ran, Rui Zhang, Weibo Sun, Oganezov Giorgi, Wenxuan Zhang, Tong Liu, Sheng Tai, Cheng Qian

**Affiliations:** Department of tumor surgery, Harbin Medical University Cancer Hospital, Harbin, Heilongjiang 150081 China

**Keywords:** SELENOP, Macrophage-endothelial crosstalk, Vascular invasion, Cholesterol metabolism, Hedgehog signaling, Breast cancer

## Abstract

**Background:**

Vascular invasion (VI) is an early event in breast cancer dissemination. However, the mechanisms by which macrophages and endothelial cells cooperate to drive this process remain incompletely understood. We aimed to identify macrophage subsets associated with VI and determine how they influence endothelial cell behavior.

**Methods:**

Single-cell RNA sequencing and spatial transcriptomic profiling and were conducted on breast cancer specimens partitioned by their vascular invasion characteristics. Multiplex immunofluorescence, coculture assays, transcriptomic, proteomic, and metabolomic profiling, molecular docking, and co-immunoprecipitation were used to characterize the SELENOP-LRP8 axis. In vivo validation was conducted using a myeloid-specific ***Selenop*** knockout model (*Selenop*^*f/f*^; *Lyz2*^*Cre*^) bearing E0771 allografts and treated with bevacizumab and/or Ebronucimab.

**Results:**

A distinct SELENOP^+^ macrophage subset was enriched in VI-positive breast tumors and exhibited close spatial proximity to endothelial cells. In vitro, macrophage-derived SELENOP enhanced endothelial migration and tube formation. Multi-omics analyses suggested that SELENOP binds endothelial LRP8, leading to cholesterol metabolic rewiring and Hedgehog pathway activation. In the mouse model, ***Selenop*** deficiency reduced tumor growth and vascularization, while combination treatment with bevacizumab and Ebronucimab produced the strongest inhibitory effect among the treatment groups.

**Conclusions:**

These findings identify a SELENOP^+^ macrophage-endothelial metabolic axis associated with breast cancer VI and support further investigation of combined anti-angiogenic and cholesterol-pathway-directed strategies in preclinical models.

**Supplementary Information:**

The online version contains supplementary material available at https://doi.org/10.1007/s00018-026-06359-y.

## Highlights

SELENOP^+^ macrophage subpopulation identified via scRNA-seq is enriched in breast cancers with vascular invasion associated with poor prognosis.

Direct SELENOP–LRP8 binding on endothelial cells triggers cholesterol metabolic reprogramming and Hedgehog pathway activation, thereby enhancing pro-angiogenic capacity.

Spatial transcriptomics and multiplex immunofluorescence confirm striking co-localization of SELENOP^+^CD68^+^ macrophages with CD31^+^ endothelial cells at the invasive front.

Selenop knockout in myeloid cells reduces M2 macrophage polarization and tumor vascularization in vivo, validating SELENOP as a functional driver of these processes.

Bevacizumab + Ebronucimab combination therapy in Selenop^f/f^; Lyz2^Cre^ mice achieve superior suppression of breast cancer VI compared with monotherapy.

**Supplementary Information:**

The online version contains supplementary material available at https://doi.org/10.1007/s00018-026-06359-y.

## Introduction

Globally, breast cancer continues to be a principal driver of oncology-related mortality, with distant metastasis serving as the chief factor underlying unfavorable patient prognosis [1]. Vascular invasion (VI), defined as tumor infiltration into the vascular system, represents an early and clinically significant step in hematogenous dissemination [2, 3]. Clinical studies have identified VI as an adverse prognostic factor across multiple solid tumors [4]. As VI develops within a complex tumor microenvironment (TME), elucidating the molecular mechanisms underlying immune-endothelial crosstalk may reveal therapeutically relevant targets [5, 6].

Within the TME, tumor‑associated macrophages (TAMs) are major contributors to tumor progression and angiogenesis, with M2‑like subsets exhibiting particularly strong pro‑angiogenic activity [7, 8]. Recent single‑cell and spatial studies have revealed substantial heterogeneity within TAM populations, suggesting diverse roles in shaping the vascular microenvironment [9, 10]. TAMs secrete multiple angiogenic mediators that promote endothelial proliferation and sprouting. In parallel, accumulating evidence indicates that endothelial metabolic states, particularly glycolysis, fatty‑acid utilization, and cholesterol homeostasis, play critical roles in regulating angiogenic switching [11, 12]. Dysregulated cholesterol flux can modify membrane organization and receptor signaling, thereby facilitating endothelial migration and vessel remodeling [13]. However, the mechanisms by which TAMs reprogram endothelial metabolism to promote angiogenesis and VI remain unclear.

SELENOP is a secreted selenoprotein that also functions as a carrier of lipid‑associated molecules [14]. Its receptor, LRP8 (also known as ApoER2), belongs to the low-density lipoprotein cholesterol (LDL) receptor family and regulates cellular lipid uptake and membrane homeostasis. In endothelial cells, LRP8 signaling has been implicated in cholesterol trafficking, membrane composition, and receptor localization, processes that collectively influence endothelial responsiveness to angiogenic stimuli [15–17]. Accordingly, SELENOP-LRP8 may regulate cholesterol flux and downstream signaling pathways, providing a biologically plausible mechanism through which macrophage‑derived SELENOP modulates endothelial metabolic states and vascular behavior.

However, it remains unclear how macrophage subsets reshape endothelial metabolic states to promote angiogenesis and VI. Consequently, we hypothesized that a distinct macrophage population in the breast cancer microenvironment engages endothelial cells through a defined ligand–receptor mechanism that remodels cholesterol-related metabolic pathways, thereby establishing an invasion‑permissive vasculature. To address this, we aimed to identify macrophage subsets associated with VI, characterize their signaling interactions with endothelial cells, and determine whether these interactions drive endothelial metabolic reprogramming relevant to angiogenic and invasive behaviors.

## Materials and methods

### Selection of human subjects

Primary breast cancer tissues suitable for surgical resection were acquired from patients undergoing therapy. Pathological verification of vascular invasion (VI) was conducted postoperatively, characterized by the identification of neoplastic cells within blood vessels lined by endothelium. During surgery, fresh tumor specimens were isolated from viable tumor zones (comprising the central tumor mass and, when accessible, the peripheral invasive border). For comparative investigations, non-neoplastic mammary tissues situated 1 to 2 cm outside the tumor boundaries, subsequently verified by histology to lack cancer cells, were retrieved from selected donors. Eligible subjects met the following requirements: high-quality tissue yield, no exposure to preoperative neoadjuvant treatment, and availability of comprehensive clinical records. Every patient provided written informed consent prior to donation. Ethical clearance for all human protocols was granted by the Institutional Ethics Committee of Harbin Medical University Cancer Hospital.

### Single-cell RNA sequencing

Resected breast tumor specimens were immediately processed into single-cell suspensions. The tissues were mechanically diced and enzymatically dissociated using a mixture of DNase I and collagenase IV at 37 °C. The cellular suspension was passed through a 40-µm nylon mesh filter, followed by incubation in red cell lysis buffer to eliminate erythrocytes. Trypan blue staining was utilized to verify cell density and viability before loading cells onto the 10x Genomics Chromium controller for single-cell capture and library construction. The sequencing cohort comprised specimens from 10 patients, evenly distributed between VI-positive (*n* = 5) and VI-negative (*n* = 5) statuses (demographics detailed in Supplementary Table 1).

Demultiplexing, barcode processing, and UMI count generation from raw FASTQ datasets were executed using Cell Ranger (10x Genomics) [18]. Downstream single-cell profiling was conducted in R using the Seurat package [19]. A total of 53,927 cells passed quality filtration across all samples, displaying similar recovery rates between the two patient groups. Cells were filtered to exclude doublets and damaged cells based on the following criteria: 200 to 6,000 expressed genes per cell and a mitochondrial read percentage under 10%. After normalization and scaling of the datasets, regression analysis was performed to mitigate bias from total UMI counts and mitochondrial gene expression. The top 2,000 variable features were selected to conduct Principal Component Analysis (PCA). Unsupervised graph-based clustering and Uniform Manifold Approximation and Projection (UMAP) were performed for visualization. Biological identities of the cell clusters were determined using reference databases alongside manual verification of classic cell-type markers.

### Spatial transcriptomics

Cryopreserved breast cancer blocks from the same 10-patient cohort (5 VI-positive, 5 VI-negative) were sectioned at a thickness of 10 μm and transferred onto 10x Genomics Visium Spatial Gene Expression slides [20]. Six representative tissue sections were selected for downstream spatial profiling. Following sectioning, tissues underwent fixation, hematoxylin and eosin (H&E) staining, high-resolution imaging, and enzymatic permeabilization to facilitate mRNA hybridization onto spatially barcoded capture spots. Libraries were synthesized following the Visium standard workflow and sequenced on an Illumina platform. Representative images shown in the figures depict high-quality areas congruent with the entire dataset.

Space Ranger software was employed to process raw sequencing reads, aligning transcripts to spatial coordinates and generating feature-spot matrices coupled with histological imagery. Data processing in R retained spots presenting a median of roughly 5,830 UMIs and 2,050 unique genes. Systemic variations across slides and sequencing batches were reconciled using the integration functions in Seurat. Finally, the spatial transcriptomic matrices were deconvoluted using paired single-cell datasets from the same patient cohort via robust cell-type decomposition (RCTD) to map the spatial distribution of distinct cell populations [21].

### Multiplex immunofluorescence

Deparaffinization and sequential rehydration via graded ethanol solutions were carried out on the FFPE specimens, followed by epitope unmasking heated in citrate buffer (pH 6.0). To block endogenous peroxidase, sections were incubated with a 3% hydrogen peroxide solution [22]. Following a blocking step with 10% normal goat serum to minimize non-specific signals, the samples were exposed overnight at 4 °C to primary antibodies against CD31 (Abmart, Cat# TA6191S), SELENOP (Santa Cruz Biotechnology, Cat# sc-376858), and CD68 (Proteintech, Cat# 66231-1-Ig). Sections were subsequently treated with HRP-conjugated secondary antibodies (anti-rabbit: Proteintech, Cat# SA00014-9; anti-mouse: Proteintech, Cat# SA00014-10) and visualized using tyramide signal amplification (TSA). For multi-target detection, sequential rounds of antigen retrieval and antibody incubation were performed. Nuclei were counterstained with DAPI (Solarbio, Cat# 422801), and cover slips were applied using antifade mounting medium prior to visualization on a multichannel fluorescence microscope.

### Lentiviral transduction

To induce macrophage differentiation, THP-1 monocytes were treated with 100 ng/mL phorbol 12-myristate 13-acetate (PMA) for 48 h. Differentiated cells were subsequently infected with lentiviral vectors containing SELENOP-targeting shRNA, a SELENOP overexpression cassette, or respective mock control vectors [23]. Infection was executed at a multiplicity of infection (MOI) of 20 in media supplemented with 8 µg/mL polybrene. The culture medium was refreshed 24 h post-infection. Selection of stably integrated cell populations was initiated 72 h later using 2 µg/mL puromycin. Validation of knockdown or overexpression efficiency was carried out via Western blot or fluorescence imaging.

### Cell culture

Cell lines, including THP-1 monocytes (ATCC, Cat# TIB-202), HUVECs (ATCC, Cat# CRL-1730), and breast cancer cell lines MDA-MB-231 (ATCC, Cat# HTB-26) and E0771 (ATCC, Cat# CRL-3461), were purchased from the American Type Culture Collection (ATCC). THP-1 cells were maintained in RPMI-1640 medium containing 10% fetal bovine serum (FBS) and 1% penicillin–streptomycin in a 37 °C incubator with 5% CO_2_. Culture of E0771 and MDA-MB-231 cells was performed in DMEM supplemented with 10% FBS. HUVECs were grown in specialized endothelial cell medium containing a 1% growth factor supplement. All cells were maintained in a humidified 5% CO_2_ at 37 °C and routinely passaged when reaching 80% confluence.

### Western blotting

Whole-cell proteins were isolated utilizing ice-cold RIPA lysis buffer. Lysates were centrifuged at 13,300 × g for 15 min at 4 °C to pellet cellular debris, and supernatant protein content was determined using a BCA protein assay kit. Proteins were denatured at 95 °C for 5 min in 4× Laemmli buffer, resolved by SDS-PAGE, and electroblotted onto PVDF membranes. Membranes were blocked with 5% skim milk in TBST buffer, followed by overnight incubation at 4 °C with primary antibodies against: SELENOP (Santa, Cat# sc-376858), ARG1 (Proteintech, Cat# 16001-1-AP), CD206 (Proteintech, Cat# 81525-1-RR), α-tubulin (Proteintech, Cat# 66603-1-Ig), LRP8 (Abcam, Cat# ab108208), VEGFA (Proteintech, Cat# 19003-1-AP), SMO (Abcam, Cat# ab235183), PLCγ1 (CST, Cat# 2822), and phosphorylated PLCγ1 (CST, Cat# 2821). After washing with TBST, the blots were probed with horseradish peroxidase (HRP)-conjugated secondary antibodies (anti-mouse: Proteintech, Cat# RGAM004; anti-rabbit: Proteintech, Cat# RGAR002) at ambient temperature for 1 h. Protein signals were subsequently detected employing an enhanced chemiluminescence (ECL) kit.

### Transwell migration assay

Polycarbonate membrane inserts featuring an 8-µm pore size, situated within 24-well plates, were employed for the cell migration evaluation [24]. Macrophage-derived conditioned media (collected after 24 h, centrifuged at 300 × g for 5 min, and filtered through 0.22-µm syringe filters) was utilized to stimulate endothelial cells. Prior to seeding, HUVECs underwent a 12-h serum starvation period, after which they were resuspended in serum-free medium at a density of 2 × 10^5^ cells/mL. A volume of 200 µL of this cell suspension was introduced into the upper chamber, while 600 µL of medium supplemented with 10% FBS was added to the lower well. After 24 h of incubation, non-migrating cells on the top of the filter were mechanically removed. The cells that successfully traversed to the bottom surface underwent fixation with 4% paraformaldehyde (PFA) and subsequent staining using 0.1% crystal violet prior to imaging. Cell numbers were enumerated from five randomly selected visual fields per insert via ImageJ. All experiments were performed in independent triplicates.

### Wound-healing assay

Endothelial cells were seeded in six-well plates and allowed to grow to complete confluence [25]. A straight physical gap was introduced into the monolayer using a sterile 200-µL pipette tip, followed by gentle washing with PBS to clear cellular debris. Cells were then maintained in low-serum or serum-free conditions to control for proliferation. Scratch closure was photographed at 0 h and 24 h in designated fields of view, and the rate of wound healing was computed via the ImageJ wound-healing plugin. These assays were replicated in three independent experiments.

### Tube-formation assay

Tube-formation assays were performed to assess endothelial angiogenic capacity [26]. Matrigel was thawed at 4 ℃, dispensed into precooled 96-well plates (50 µL/well), and incubated at 37 ℃ for 30–45 min. HUVECs were resuspended in serum-free or conditioned medium (5 × 10^4^ cells/mL) and plated onto the Matrigel (100 µL/well). After 4–6 h of incubation, tube-like structures were imaged and quantified using the ImageJ angiogenesis analyzer plugin, including total tube length, branch points, and mesh formation. Each experiment was repeated at least three times.

### Multi-omics profiling of endothelial cells

#### Transcriptomic profiling

Endothelial cells were collected, washed twice with cold PBS, and directly lysed in TRIzol reagent (Invitrogen). Purification of total RNA was conducted using the RNeasy Mini Kit (Qiagen), with RNA integrity assessed on an Agilent 2100 Bioanalyzer. Only RNA specimens demonstrating an RNA integrity number (RIN) greater than 7.0 were utilized for subsequent library construction. The enrichment of poly(A)+ transcripts was achieved via oligo-dT-attached magnetic beads, followed by library preparation utilizing the NEBNext Ultra II RNA Library Prep Kit (NEB). Sequencing was performed on the Illumina NovaSeq 6000 instrument using a 150-bp paired-end configuration. Trimming of adapter sequences and low-quality reads was performed via Trimmomatic, followed by genomic alignment to the GRCh38 assembly using STAR. Gene expression quantification was performed with featureCounts. Multi-level quality checks verified mapping rates exceeding 85%, acceptable read duplication levels, and even gene body coverage. Normalization was executed via the variance-stabilizing transformation method within DESeq2. The DESeq2 package was employed to screen for differentially expressed genes (DEGs). To control the false discovery rate (FDR), P-values were adjusted using the Benjamini–Hochberg method, with a significance threshold set at FDR < 0.05.Functional analysis was conducted using Gene Set Enrichment Analysis (GSEA) and the ReactomePA package.

#### Proteomic profiling

After PBS washing, cell pellets were lysed using SDS-containing buffer, and protein concentrations were measured using a BCA assay kit. Reduction of disulfide bonds was achieved with 10 mM DTT at 56 °C for 30 min, followed by alkylation in the dark using 20 mM iodoacetamide. Enzymatic digestion was performed overnight with sequencing-grade trypsin (Promega; 1:50 enzyme-to-protein ratio) via the filter-aided sample preparation (FASP) protocol. The resulting peptide mixtures were desalted using C18 microcolumns and analyzed on an EASY-nLC 1200 nano-flow system online-coupled to an Orbitrap Fusion Lumos mass spectrometer (Thermo Fisher). High-resolution MS1 scans (120,000 resolution) were acquired, and precursor ions were fragmented via HCD to generate MS/MS spectra. Mass spectrometry files were searched using MaxQuant against the UniProt database, maintaining a 1% false discovery rate (FDR) threshold for peptides and proteins. Label-free quantification (LFQ) values were log2-transformed, and quantile normalization was applied. Non-biological batch variations were adjusted via the ComBat algorithm (sva package). Proteins containing over 50% missing values across conditions were filtered out. Differentially abundant proteins were identified using moderated t-tests (FDR < 0.05). Functional pathways were analyzed using DAVID and Reactome resources. Quality controls verified robust trypsin digestion efficiency, stable total ion chromatograms, high mass accuracy (< 3 ppm), and low technical variability (CV < 20%).

#### Metabolomic profiling

To eliminate extracellular metabolites, cells were washed twice with ice-cold PBS, immediately snap-frozen in liquid nitrogen, and extracted with a cold extraction solvent mixture (methanol/acetonitrile/water, 2:2:1 v/v). Following vortexing, the mixture was incubated at -20 °C for 1 h and cleared by centrifugation at 14,000 × g for 15 min at 4 °C. The supernatant fraction was dried under a stream of nitrogen gas and reconstituted in 50% acetonitrile. Metabolite profiling was conducted on an UltiMate 3000 UHPLC system coupled to a Q Exactive Plus MS (Thermo Fisher) operating in electrospray ionization (ESI) positive and negative modes. LC separation was carried out using a Waters HSS T3 column (2.1 × 100 mm, 1.8 μm). Raw metabolomic runs were processed in XCMS for peak identification, alignment, and grouping. Probabilistic quotient normalization (PQN) was used to correct for variations in total cell number. Features with missing values in more than 20% of samples were discarded, while the remaining missing values were imputed using the k-nearest neighbors (KNN) algorithm. Quality assurance was monitored by running pooled QC samples after every ten injections, ensuring stable retention times (drift < 0.1 min), high mass accuracy (< 5 ppm), symmetrical peaks, and a technical CV under 20%. Gene‑level counts were generated using featureCounts. Quality control included assessment of mapping rate (> 85%), read duplication, and gene body coverage. Data normalization was performed using DESeq2 variance‑stabilizing transformation. Differential expression was assessed using DESeq2 with Benjamini–Hochberg correction (false discovery rate [FDR] < 0.05). Furthermore, enrichment analysis was conducted using GSEA and ReactomePA.

#### Immunoaffinity purification-mass spectrometry

Protein samples were denatured at 95 ℃ for 5 min, reduced with tris(2-carboxyethyl)phosphine, and alkylated with chloroacetamide at 95 ℃ for 10 min. After the addition of hydroxypropyl-β-cyclodextrin, proteins were digested with trypsin overnight at 37 ℃ [27]. Digestion was stopped with trifluoroacetic acid, and peptides were purified using SDB-RPS desalting columns. Peptides were separated on an UltiMate 3000 RSLCnano system and analyzed on a Q Exactive HF mass spectrometer in data-dependent acquisition mode. Raw mass spectrometry files were processed with MaxQuant (version 2.2.0.0) against the human UniProt database. Database searches included carbamidomethylation as a fixed modification, oxidation and deamidation as variable modifications, a maximum of two missed tryptic cleavages, and a 1% FDR at the peptide and protein levels.

#### Molecular docking

Molecular docking was performed using protein structures predicted by AlphaFold [28]. High-confidence models were prepared by removing heteroatoms, adding hydrogen atoms, and assigning atomic charges. Ligands were energy-minimized before docking. Docking simulations were performed using AutoDock Vina with the search grid centered on the predicted binding pocket. The highest-ranking poses were evaluated based on predicted binding energy and intermolecular contacts.

#### Metabolic flux analysis

Cells were incubated with [13] C-labelled glucose in isotope-tracing medium. After the designated time points, metabolism was quenched with cold methanol-water, and metabolites were extracted using a methanol-chloroform-water system. Polar fractions were analyzed for central carbon metabolism, whereas organic extracts were used for lipid profiling. Metabolites were measured by liquid chromatography-tandem mass spectrometry using hydrophilic interaction chromatography for polar metabolites and reversed-phase C18 chromatography for lipids. Isotopologue distributions were analyzed using XCalibur and MAVEN, and metabolic fluxes were estimated using INCA software.

#### Co-immunoprecipitation

Cells were lysed in radioimmunoprecipitation assay buffer containing protease inhibitors and centrifuged at 12,000 × g for 15 min at 4 ℃. Lysates were quantified by bicinchoninic acid assay, pre-cleared with protein A/G magnetic beads, and incubated overnight with primary antibodies or isotype-matched immunoglobulin G controls. Immune complexes were captured using protein A/G beads, washed four times, eluted in 2× Laemmli buffer, and analyzed by Western blotting.

#### Calcium Imaging

Intracellular calcium dynamics were monitored by loading HUVECs with the calcium-sensitive dye Fluo-4 AM (Solarbio, Cat# CA1190) following the manufacturer’s protocol. After loading and washing, cells were allowed to de-esterify in fresh medium. Imaging was performed on an inverted fluorescence microscope with a camera. For Fluo‑4, excitation was at 488 nm and emission collected at 525 nm.Time‑lapse images were acquired at regular intervals. Regions of interest (ROIs) were drawn over individual cells, and background fluorescence was subtracted.

#### Genetically engineered mouse models

Transgenic mice were generated by Cyagen Biosciences using CRISPR/Cas9 technology. ***Selenop*** ^flox/flox^ mice were crossed with ***Lyz2***-^Cre^ mice to generate myeloid-specific ***Selenop*** conditional knockout mice. Genotypes were confirmed by polymerase chain reaction. The animals were maintained in a specific pathogen-free (SPF) facility, receiving food and water ad libitum. Ethical approval for all in vivo protocols was granted by the Animal Ethics and Welfare Committee of Harbin Medical University. For tumor studies, 4–6-week-old wild-type and conditional-knockout C57BL/6 mice were inoculated subcutaneously with E0771 cells. Ebronucimab was administered at 15 mg/kg once weekly until the experimental endpoint. Bevacizumab was administered at 15 mg/kg twice per week in mouse models.

#### Flow cytometry

Mouse tumors were harvested and processed into single-cell suspensions via mechanical dissociation and filtration through 70-µm cell strainers [29]. Erythrocytes were eliminated using ammonium-chloride-potassium (ACK) lysing buffer, and the remaining cells were washed with PBS containing 2% FBS. Cells were incubated with fluorochrome-conjugated surface antibodies in the dark at 4 °C for 30 min. For intracellular protein detection, cells were fixed, permeabilized, and stained. Data acquisition was performed on a BD FACS Celesta flow cytometer, and analysis was executed via FlowJo software.

#### Statistical analysis

All quantitative results are depicted as the mean ± standard deviation (SD), derived from at least three biological replicates. Statistical significance between two experimental conditions was determined via an unpaired, two-tailed Student’s t-test. For comparisons involving three or more groups, a one-way ANOVA combined with Tukey’s multiple comparison test was applied. Regarding single-cell RNA sequencing data, differentially expressed genes were identified using the Wilcoxon rank-sum algorithm within the Seurat framework, with P-values adjusted for multiple testing [30]. Statistical significance for all experiments was set at *P* < 0.05. Proteomic and metabolomic data were analyzed using multivariate approaches, including principal component analysis and partial least squares discriminant analysis. Features with a fold change > 2 and an FDR < 0.05 were considered significantly altered. Analyses were performed in R (version 4.1.0) or GraphPad Prism (version 9.0), with *P* < 0.05 considered significant [31].

## Results

### Single-cell RNA sequencing reveals a distinct TME in breast cancers with VI

**Fig. 1:**
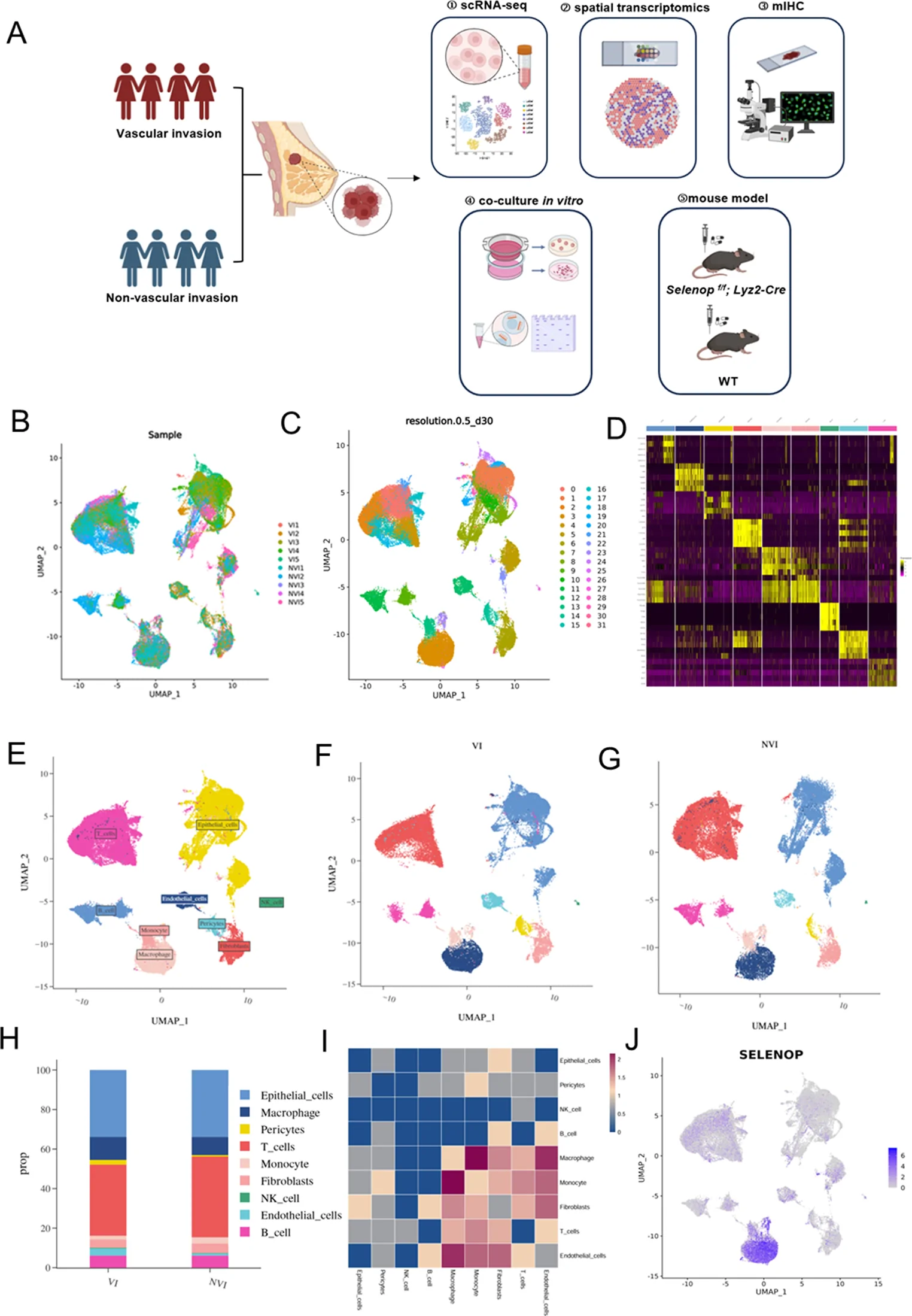
Single-cell transcriptomics delineates the tumor microenvironment in breast cancer with vascular invasion. (**A**). Schematic diagram of the experimental procedure. (**B**–**C**). Uniform manifold approximation and projection (UMAP) visualization colored by samples and single-cell clusters. (**D**). Heatmap of classic gene markers for cell type annotation. (**E**). Cell type annotation visualized by UMAP. (**F**–**G**). UMAP visualization of tumor microenvironment heterogeneity between groups. (**H**). Bar plot of cell type proportions to show the different cell type in breast cancer tissues with and without VI. (**I**). Heatmap of cell-cell communication to display the interaction patterns among different cell types in group VI. (J). An overview of SELENOP expression patterns in various cell compartments is illustrated in the UMAP plot

To characterize the TME associated with VI, breast cancer specimens were stratified according to VI status. The overall study design is summarized in Fig. 1A. We performed single-cell sequencing on 5 samples per group. Single-cell RNA sequencing of dissociated tumors followed by quality control, dimensionality reduction, and unsupervised clustering identified nine major cell populations: macrophages, pericytes, monocytes, fibroblasts, T cells, natural killer cells, endothelial cells, epithelial cells, and B cells (Figs. 1B–E). Comparative analysis demonstrated that macrophages, endothelial cells, and pericytes were enriched in VI-positive samples (Figs. 1F–H), implicating these populations in the biology of VI.

CellChat analysis further indicated stronger and more complex macrophage-endothelial communication networks in the VI-positive group (Fig. 1I). In the UMAP plot, we examined the expression of our subsequent target gene SELENOP and found that although SELENOP was also expressed in other cell types, its expression was highest and most prominent in macrophages (Fig. 1J). Collectively, these data provide a high-resolution map of the breast cancer microenvironment and nominate macrophage-endothelial interactions as a candidate axis in VI pathogenesis.

### SELENOP^+^ macrophages are enriched in breast cancer with VI

**Fig. 2:**
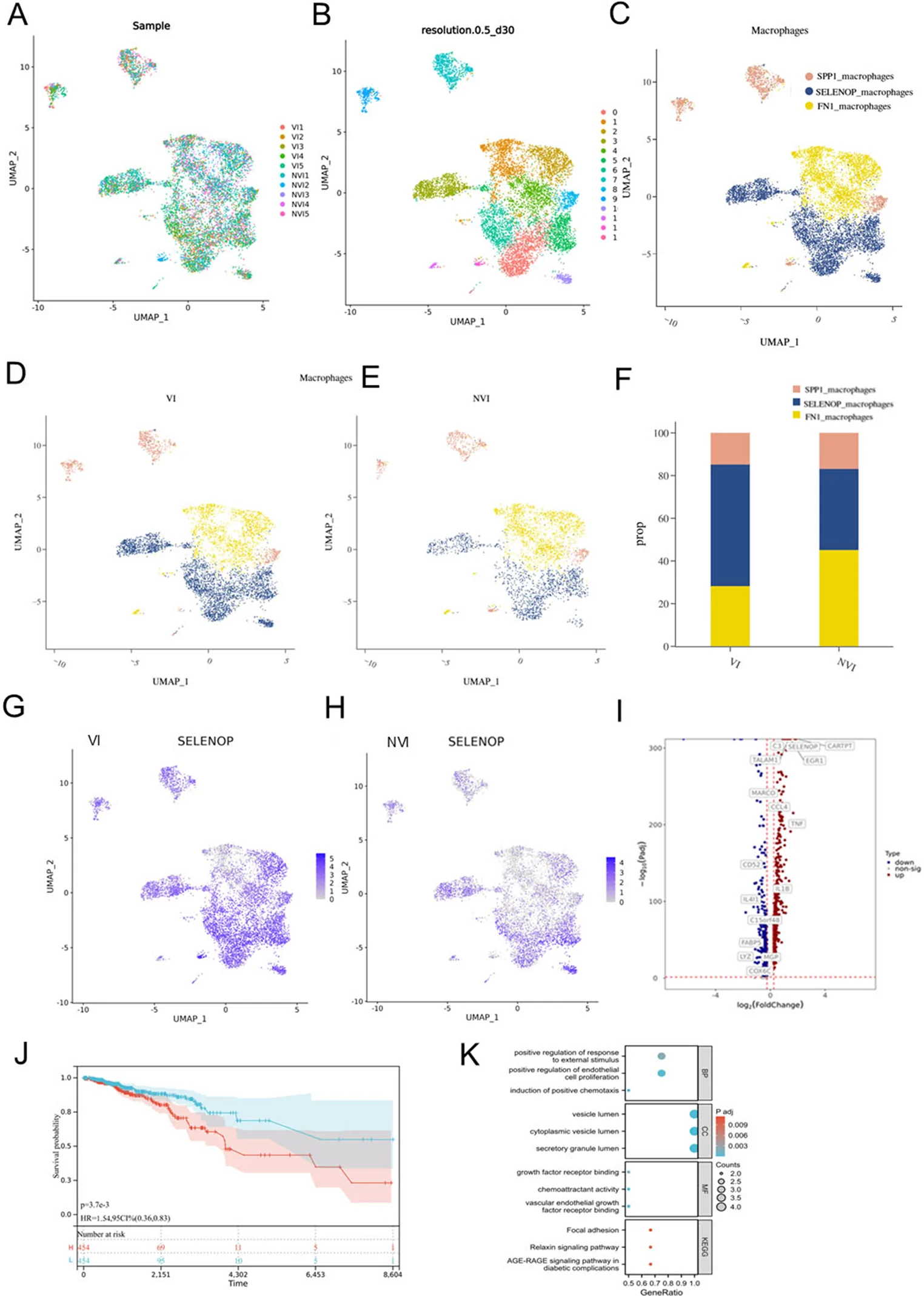
A distinct population of SELENOP^+^ macrophages was identified in the vascular invasion group of breast cancer. (**A**–**B**). UMAP plot showing macrophage sub clustering, colored by sample identity and cell cluster. (**C**). UMAP to show annotation of macrophage subclusters based on classic markers of macrophage. (**D**–**E**). Comparative analysis of macrophage subclusters between groups visualized by UMAP. (**F**). Quantitative analysis of macrophage subclusters between VI and NVI groups. (**G**–**H**). The UMAP plot shows the differences in the expression of SELENOP in breast cancer tissues with and without VI. (**I**). Volcano plot to show the different expression genes of macrophages in two groups. Genes Upregulated in group VI are highlighted in red. (**J**). Kaplan-Meier curve demonstrating the prognostic value of SELENOP+ macrophages in the TCGA breast cancer cohort. The red curve represents hi 591 gh expression of SELENOP+ macrophages. (**K**). Bubble plot to show functional enrichment analysis of SELENOP+ macrophages

To examine macrophage heterogeneity in greater detail, we re-clustered the macrophage compartment from the full single-cell dataset. Three transcriptionally distinct subsets were identified: FN1^+^ macrophages, SPP1^+^ macrophages, and SELENOP^+^ macrophages (Figs. 2A–C). Comparison between VI-positive and VI-negative tumors demonstrated marked enrichment of SELENOP^+^ macrophages in the VI-positive group (Figs. 2D–F).

Differential expression analyses further identified SELENOP as one of the most upregulated genes in macrophages from VI-positive tumors (Figs. 2G–I). Genes involved in antigen presentation and endothelial regulation were also enriched, supporting a role in vascular crosstalk. In the TCGA breast cancer cohort, the inferred SELENOP⁺ macrophage signature was significantly associated with overall survival (Fig. 2J). Functional enrichment analysis demonstrated overrepresentation of pathways related to endothelial proliferation and vascular endothelial growth factor receptor binding (Fig. 2K), consistent with a pro-angiogenic phenotype.

### Endothelial cells in the VI group display a lipid-metabolic transcriptional program

**Fig. 3:**
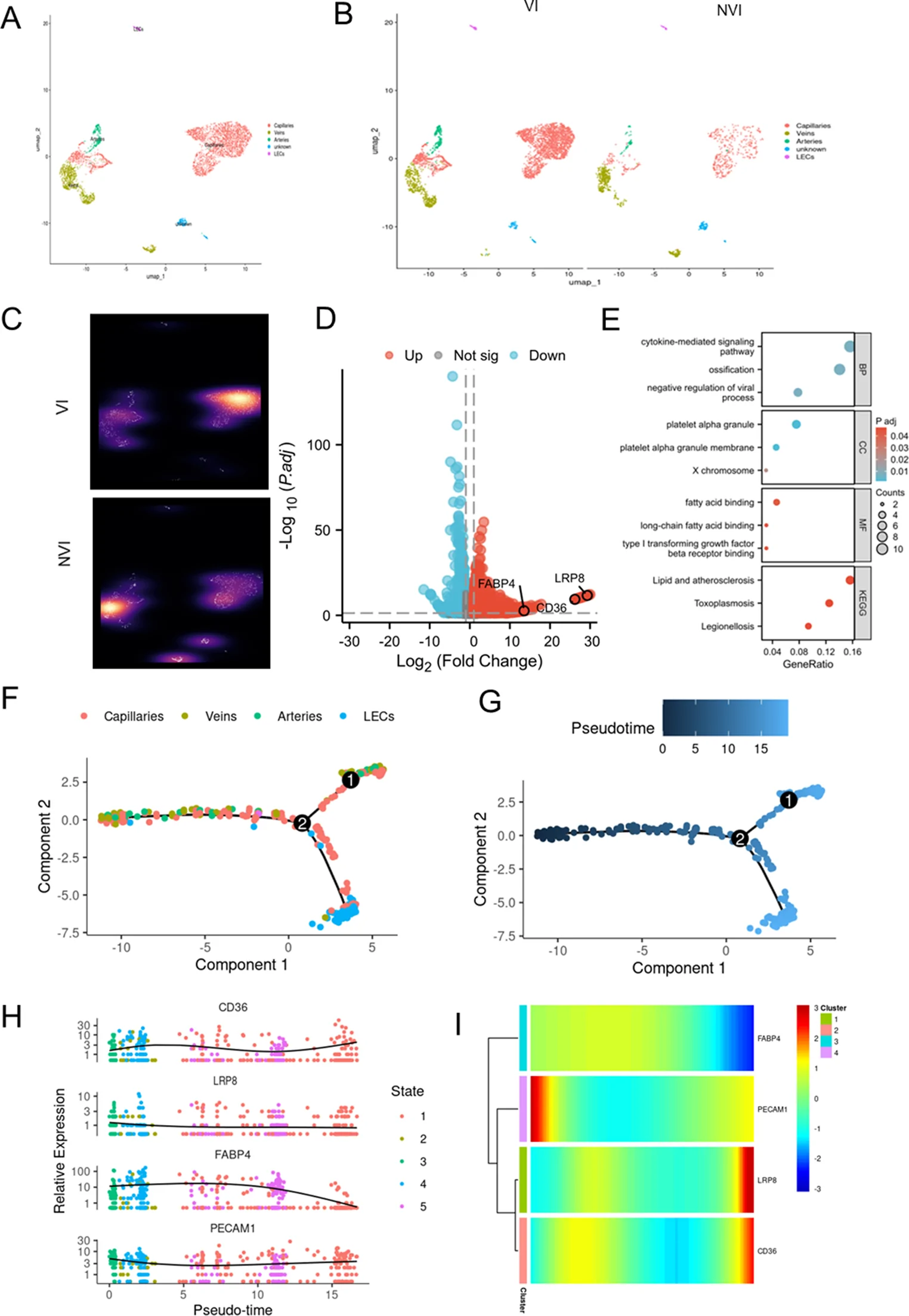
Comparative Analysis of Endothelial Cell Divergence Between Groups. (**A**). Single-cell annotation of endothelial cells. (**B**). Atlas of endothelial cell divergence between VI and NVI groups in breast cancer. (**C**). Comparative analysis of vascular density distribution across different vessel types between two groups. (**D**). Volcano plot of differentially expressed genes in endothelial cells between two groups. Genes Upregulated in group VI are highlighted in red. (**E**). Bubble plot to show pathway enrichment analysis in vascular Endothelial Cells. (**F**–**G**). Analysis of the temporal trajectory of vascular endothelial cells. (**H**–**I**). The temporal changes of the marker genes

To investigate endothelial heterogeneity in relation to VI, endothelial cells were subsetted and re-clustered. Based on canonical markers, endothelial cells were classified into arterial, venous, capillary, and lymphatic subtypes (Fig. 3A). Capillary endothelial cells were comparatively enriched and transcriptionally distinct in VI-positive tumors (Figs. 3B, C).

Differential expression analysis revealed increased expression of lipid metabolism-related genes, including *CD36*, *FABP4*, and *LRP8*, in capillary endothelial cells from VI-positive samples (Fig. 3D). Pathway enrichment analysis indicated fatty acid binding, long-chain fatty acid transport, and lipid droplet organization (Fig. 3E), suggesting metabolic rewiring toward increased lipid handling.

Pseudotime analysis using Monocle3 placed capillary endothelial cells near the root of the endothelial trajectory (Figs. 3F, G). Expression levels of *CD36*, *LRP8*, and *FABP4* peaked early in pseudotime and declined with progression toward venous or arterial states, whereas *PECAM1* remained comparatively stable (Figs. 3H, I). These findings suggest that lipid metabolism may be particularly important for capillary endothelial plasticity during vascular remodeling.

### Spatial transcriptomics localizes SELENOP⁺ macrophages adjacent to endothelial cells

**Fig. 4:**
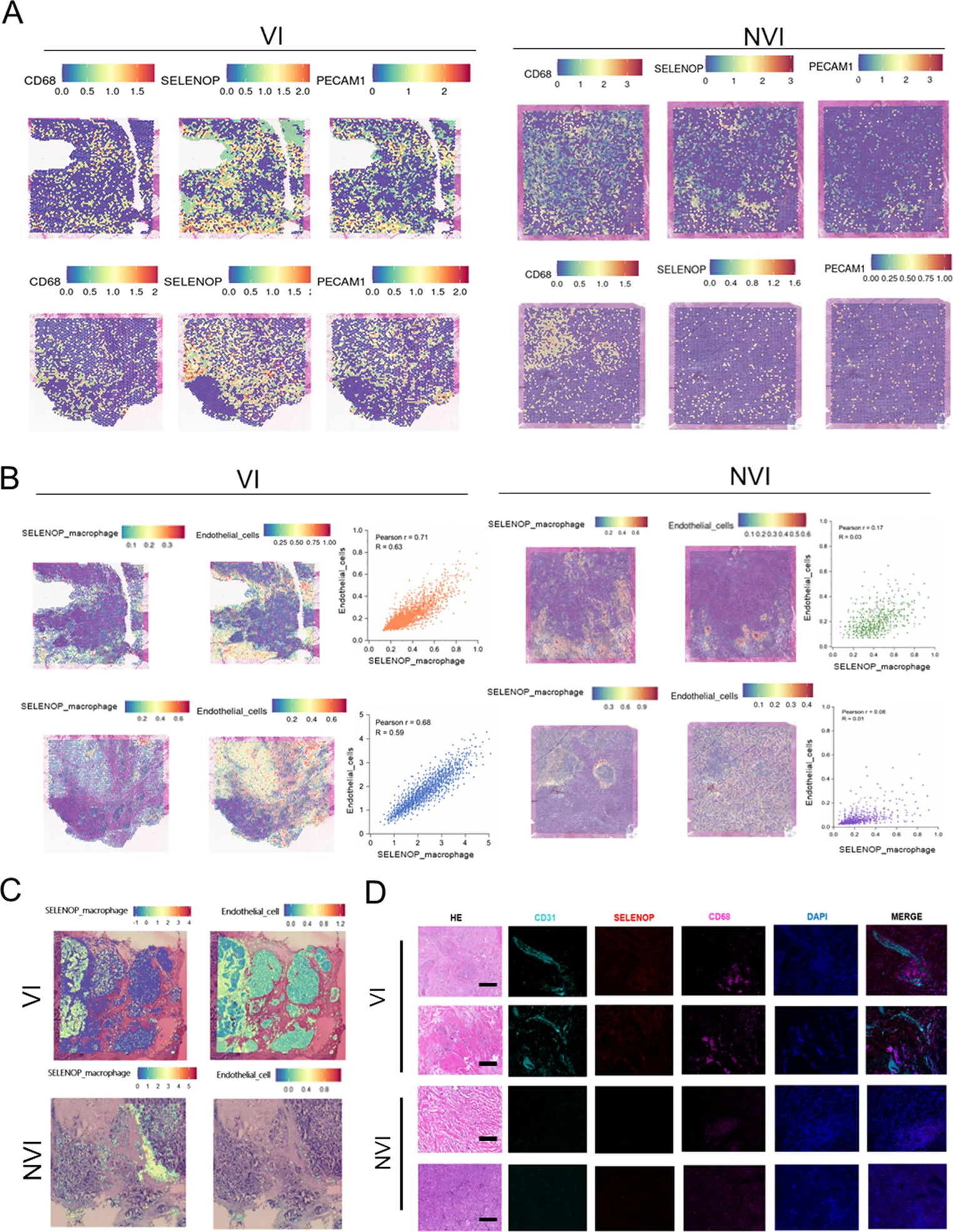
Spatial transcriptomics and multiplex immunofluorescence identify spatial co-localization of SELENOP^+^ macrophages with endothelial cells. (**A**). Spatial visualization of transcriptomic marker genes. (**B**). Spatial transcriptomics reveals co-localization between two cellular subpopulations. (**C**). Spatial co-localization of two cellular subpopulations visualized by Visium HD technology. (**D**). Representative multiplex immunofluorescence image to show the infiltration of SELENOP+ macrophage and CD31 indicate the vascular density between VI and NVI groups. Scale bar = 0.1 mm

To determine whether the interactions identified by single-cell profiling were supported at the spatial level, we performed spatial transcriptomic analysis of breast cancer sections from the same cohort. In VI-positive tumors, *CD68*, *SELENOP*, and *CD31* indicated overlapping spatial distributions (Fig. 4A). Integrating single-cell and spatial data using robust cell type decomposition confirmed significant co-localization of SELENOP⁺ macrophages and endothelial cells in VI-positive samples (Fig. 4B).

Visium HD profiling of one VI-positive tumor reproduced this pattern at higher spatial resolution (Fig. 4C). Multiplex immunofluorescence in independent human specimens further confirmed that SELENOP^+^*CD68*^*+*^ cells were frequently located adjacent to CD31^+^ endothelial structures, particularly at invasive fronts and within areas of active vascular remodeling (Fig. 4D). These observations support a direct spatial relationship between SELENOP^+^ macrophages and endothelial cells in VI-positive breast cancer.

### SELENOP^**+**^ macrophages enhance endothelial pro-angiogenic activity in vitro

**Fig. 5:**
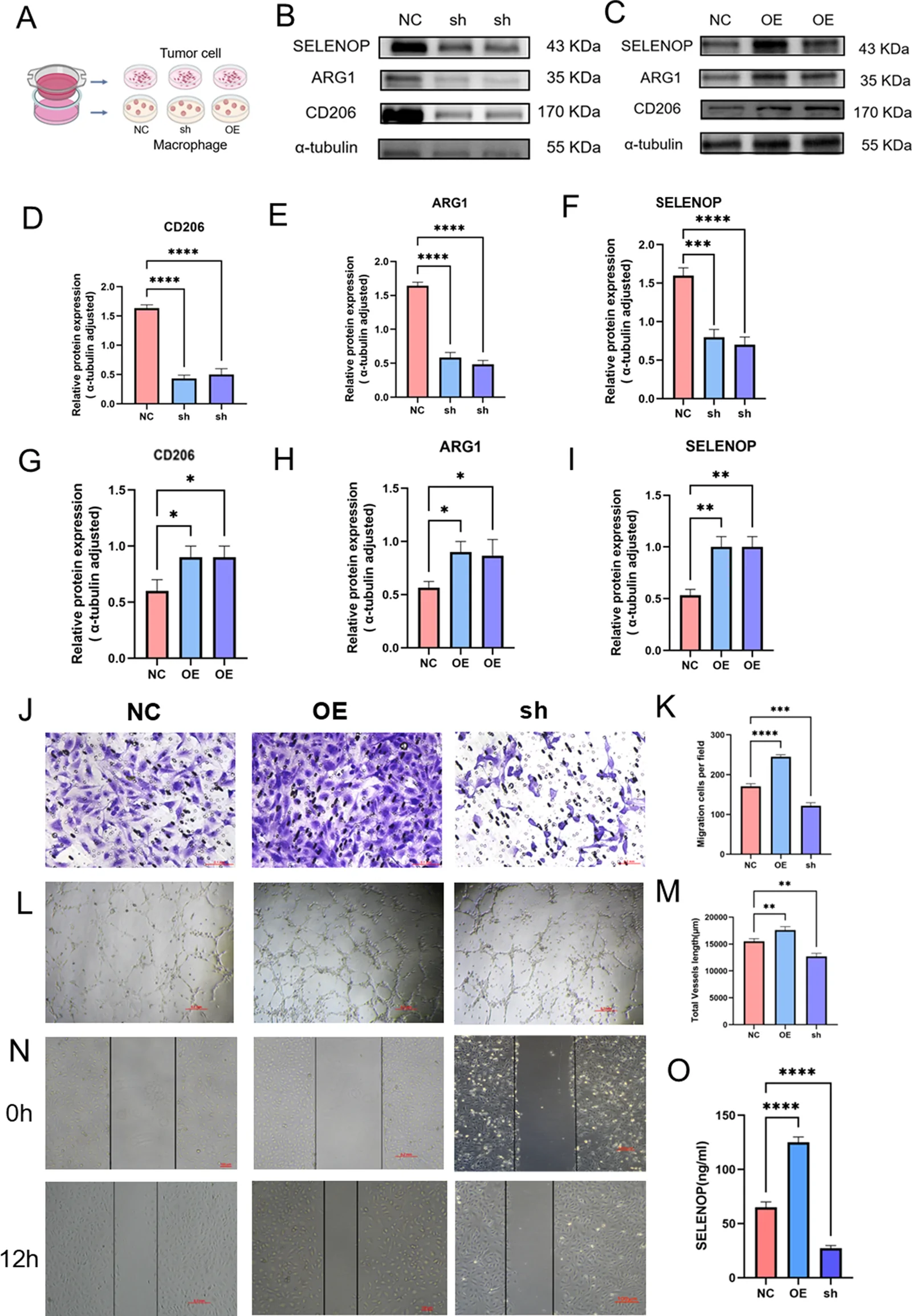
In vitro experiments demonstrate that SELENOP^+^ macrophages drive M2 polarization and promote the pro-angiogenic capacity. (**A**). Schematic illustration of the co‑culture system consisting of tumor cells and macrophages. Macrophages were transduced with negative control (NC), short hairpin RNA‑mediated knockdown of SELENOP (sh), or SELENOP overexpression (OE) constructs, as indicated, and then co‑cultured with tumor cells to evaluate the functional role of macrophage‑derived SELENOP in the tumor microenvironment. Abbreviations used in the schematic are defined as follows: NC, negative control; sh, short hairpin RNA (knockdown); OE, overexpression. (**B**). Protein-level validation shows SELENOP knockdown impairs M2 polarization program. (**C**). Western blot analysis demonstrated that SELENOP overexpression led to increased expression levels of M2 polarization markers. (**D**–**I**). Statistical analysis of marker expression levels. Error bars show the mean ± SD. P values were determined by two-tailed ordinary one-way ANOVA with Student’s t-test. (* *P* < 0.05, ** *P* < 0.01, *** *P* < 0.001, ****P < 0.0001). (**J**–**K**). Transwell assay showing the effect of co‑culture with SELENOP⁺ macrophages on endothelial cell migration. Data are based on three independent biological replicates, with five randomly selected fields quantified per condition. Scale bar = 0.1 mm. Error bars represent mean ± SD. (* P < 0.05, ** P < 0.01, *** P < 0.001, **** P < 0.0001). (**L**–**M**). Angiogenesis assays demonstrating that SELENOP⁺ macrophages promote endothelial tube formation. Quantification was performed from three biological replicates, analyzing five fields per sample. Scale bar = 0.1 mm. Error bars represent mean ± SD. (* P < 0.05, ** P < 0.01, *** P < 0.001, **** P < 0.0001). (**N**). Wound‑healing assay showing enhanced endothelial migration upon co‑culture with SELENOP⁺ macrophages. Data were obtained from three biological replicates, with five imaging fields analyzed under each condition. Scale bar = 0.1 mm. Error bars represent mean ± SD. (* P < 0.05, ** P < 0.01, *** P < 0.001, **** P < 0.0001) (**O**). SELENOP⁺ macrophages actively release SELENOP into the extracellular space. Error bars show the mean ± SD. (* P < 0.05, ** P < 0.01, *** P < 0.001, ****P < 0.0001)

To validate the functional relevance of SELENOP^+^ macrophages, we established lentiviral gain- and loss-of-function models in macrophages and examined their effects in coculture systems (Fig. 5A). SELENOP knockdown reduced expression of M2 markers ARG1 and CD206. In contrast, SELENOP overexpression increased both markers (Figs. 5B–I), indicating that SELENOP promotes an M2-like phenotype.

We next examined the effects of these macrophages on endothelial function. Endothelial cells exposed to SELENOP-deficient macrophages exhibited reduced migration in Transwell assays, impaired tube formation on Matrigel, and slower wound closure. In contrast, exposure to SELENOP-overexpressing macrophages enhanced each of these phenotypes (Figs. 5J–N). ELISA confirmed that SELENOP was secreted into macrophage-conditioned medium and that extracellular SELENOP abundance tracked with the functional effects observed (Fig. 5O). Collectively, these data indicate that SELENOP^+^ macrophages promote endothelial migration and angiogenic behavior in vitro.

### Coculture with SELENOP^+^ macrophages induces metabolic reprogramming in endothelial cells

To define the mechanisms underlying these pro-angiogenic effects, we performed integrated transcriptomic, proteomic, metabolomic, and co-immunoprecipitation mass spectrometry analyses in endothelial cells cocultured with SELENOP^+^ macrophages versus control macrophages.

**Fig. 6:**
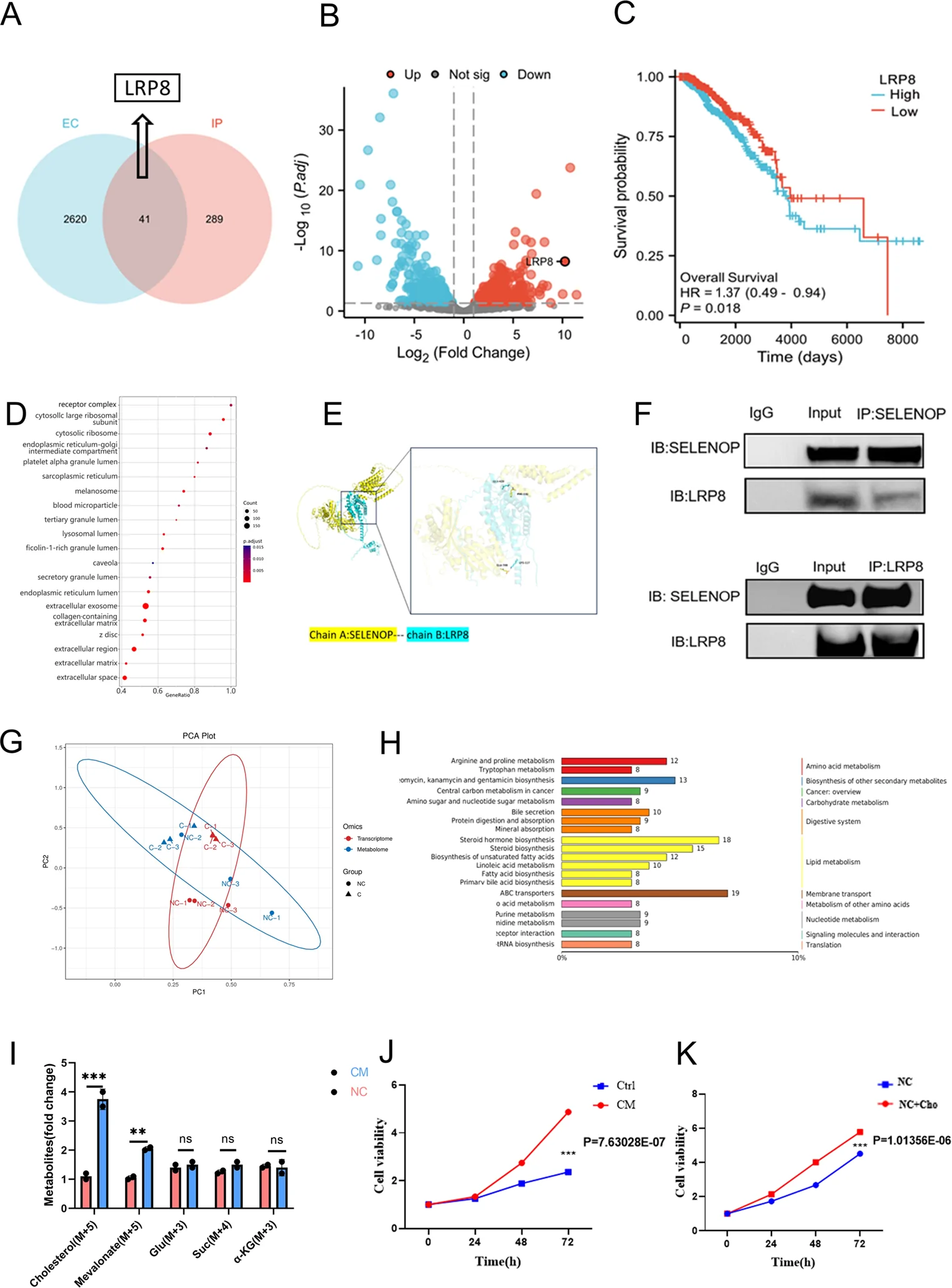
SELENOP^+^ macrophages drive metabolic reprogramming in endothelial cells. (**A**). Venn diagram of EC transcriptomic (RNA‑seq, *n* = 2,620 DEGs) and proteomic (IP‑MS, *n* = 289 SELENOP‑interacting proteins) profiles after co‑culture with SELENOP⁺ macrophages. Overlap: 41 shared candidates, with LRP8 highlighted as a convergent target. (**B**). Transcriptomic differences between co-cultured and normal endothelial cells. Upregulated genes in the co-culture group are highlighted in red. (**C**). Survival analysis of LRP8 in TCGA database breast cancer cohort. Blue curve represents the survival of patients with high LRP8 expression. (**D**). Functional enrichment analysis of LRP8. (**E**). Molecular docking predicts physical interaction between SELENOP and LRP8. The molecular structure in yellow corresponds to SELENOP, while the blue part corresponds to LRP8. (**F**). Co-immunoprecipitation confirms the interaction between SELENOP and LRP8. (**G**). PCA visualization of metabolomic and transcriptomic analysis between experimental groups and control group. (**H**). Bar plot to show the enrichment analysis of metabolic pathways. (**I**). Metabolic flux analysis revealed elevated endothelial cholesterol content in co-culture models. (**J**). CCK-8 assay demonstrated significantly enhanced endothelial cell viability in co-culture conditions. Statistical significance was assessed by one‑way ANOVA followed by Tukey’s multiple‑comparison test. (* *P* < 0.05, ** *P* < 0.01, *** *P* < 0.001, ****P < 0.0001). (**K**). Exogenous cholesterol enhances the proliferative capacity of endothelial cells One‑way ANOVA with Tukey’s post‑hoc test was used for statistical analysis. (* *P* < 0.05, ** *P* < 0.01, *** *P* < 0.001, ****P < 0.0001)

These analyses identified LRP8 as a candidate SELENOP-interacting receptor in endothelial cells (Figs. 6A, B). In the TCGA cohort, high *LRP8* expression was associated with poorer overall survival (Fig. 6C). Functional enrichment analyses linked LRP8-associated programs to receptor complex activity, platelet adhesion, and hemodynamic regulation (Fig. 6D). Molecular docking predicted a stable SELENOP-LRP8 interaction, and reciprocal co-immunoprecipitation supported direct binding (Figs. 6E, F).

Unbiased metabolomic profiling indicated clear separation between endothelial cells exposed to SELENOP^+^ macrophages and controls (Fig. 6G). Sterol metabolism and lipid transport were among the most significantly altered pathways (Fig. 6H). Isotope-tracing experiments demonstrated increased intracellular cholesterol in endothelial cells following coculture with SELENOP^+^ macrophages (Fig. 6I). These metabolic changes were accompanied by increased endothelial viability, which was further enhanced by cholesterol supplementation and attenuated by cholesterol depletion (Figs. 6J, K). Collectively, these findings support a model in which macrophage-derived SELENOP engages endothelial LRP8 and promotes cholesterol-centered metabolic reprogramming.

## SELENOP-LRP8 signaling promotes pro-angiogenic activity through Hedgehog pathway modulation

**Fig. 7:**
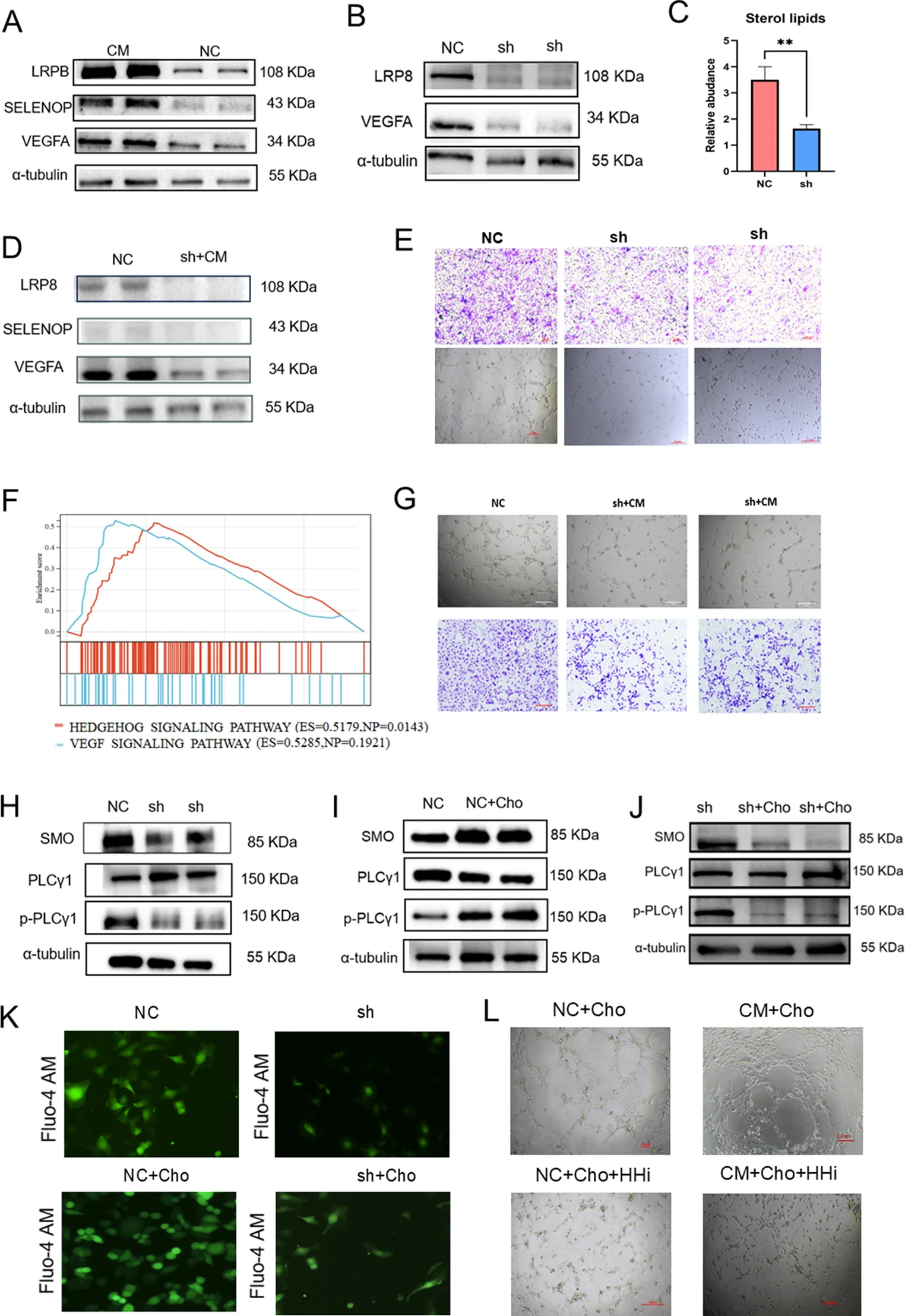
SELENOP^+^ macrophages promote the pro-angiogenic capacity by binding to the LRP8 receptor on endothelial cells. (**A**). Protein analysis in co-cultured endothelial cells. (**B**). Protein profiling in LRP8-knockdown endothelial cells. (**C**). Targeted lipidomics Analysis of LRP8-knockdown versus normal endothelial cells. (**D**). Protein analysis in co-cultured endothelial cells which LRP8 knockdown. (**E**). Functional validation of LRP8 knockdown in endothelial cells using Transwell and angiogenesis assays. (**F**). GSEA enrichment plot of co-cultured endothelial cells. (**G**). Detection of tube formation ability and migration ability of endothelial cells after co-culture. (**H**). Hedgehog pathway alterations in LRP8-knockdown endothelial cells. (**I**). Hedgehog pathway alterations in normal endothelial cells with added cholesterol. (**J**). Western blot of LRP8‑knockdown cells treated with or without cholesterol. (**K**). Fluo‑4 AM live‑cell imaging shows reduced intracellular Ca²⁺ fluorescence intensity following LRP8 knockdown. Cholesterol supplementation increases Ca²⁺ influx in control endothelial cells (NC + Cho) but has minimal effect in LRP8‑deficient cells (sh + Cho), indicating impaired cholesterol‑dependent signaling. Quantification is based on three independent biological replicates, with five randomly selected fields analyzed per condition. (**L**). Cholesterol enhances tube formation in control endothelial cells but not in LRP8‑deficient cells, and Hedgehog inhibition blocks cholesterol‑induced angiogenesis. Data were obtained from three biological replicates, quantifying five microscopic fields per condition. Scale bar = 100 μm

To define downstream signaling, we examined endothelial responses to SELENOP-LRP8 engagement. Coculture with SELENOP^+^ macrophages led to the accumulation of SELENOP protein in endothelial cells, and increased expression of LRP8 and VEGFA (Fig. 7A). Stable knockdown of *LRP8* reduced VEGFA expression and impaired endothelial migration and tube formation (Figs. 7B, E).

When *LRP8*-deficient endothelial cells were exposed to SELENOP^+^ macrophages, the pro-angiogenic effects observed in control cells were lost. SELENOP accumulation and VEGFA upregulation were not restored, and migration and tube-formation defects persisted (Figs. 7D, G), indicating that LRP8 is required for signal transmission from SELENOP^+^ macrophages.

Targeted lipidomics indicated that *LRP8* knockdown reduced sterol lipid species (Fig. 7C). Gene set enrichment analysis highlighted Hedgehog and VEGF signaling as major downstream programs (Fig. 7F). To clarify how macrophage‑derived SELENOP influences endothelial metabolic signaling, we examined key downstream molecules following LRP8 knockdown and evaluated whether cholesterol supplementation could compensate for impaired pathway activity. Western blot analysis showed that LRP8 knockdown markedly reduced SMO expression and diminished PLCγ1 phosphorylation, indicating suppression of the Hedgehog signaling axis (Fig. 7H). Importantly, cholesterol supplementation (+ Cho) increased SMO and p‑PLCγ1 abundance in control endothelial cells; however, this metabolic rescue effect was not observed when LRP8 was silenced. In LRP8‑deficient cells, cholesterol failed to restore either SMO or PLCγ1 phosphorylation (Fig. 7I, J), demonstrating that LRP8 is required for cholesterol‑dependent activation of this signaling pathway.

Consistent with these molecular findings, Fluo‑4 AM imaging revealed that LRP8 knockdown substantially weakened intracellular Ca²⁺ signals, and cholesterol supplementation did not improve Ca²⁺ influx under these conditions (Fig. 7K). In tube‑formation assays, cholesterol enhanced endothelial tubulogenesis in control cells, whereas LRP8 knockdown abolished this pro‑angiogenic response. Moreover, Hedgehog inhibition further suppressed tube formation in cholesterol‑treated control cells but had minimal additional effect in LRP8‑deficient cells (Fig. 7L).

### Combined bevacizumab and Ebronucimab treatment reduces tumor growth and vascular features in vivo

**Fig. 8:**
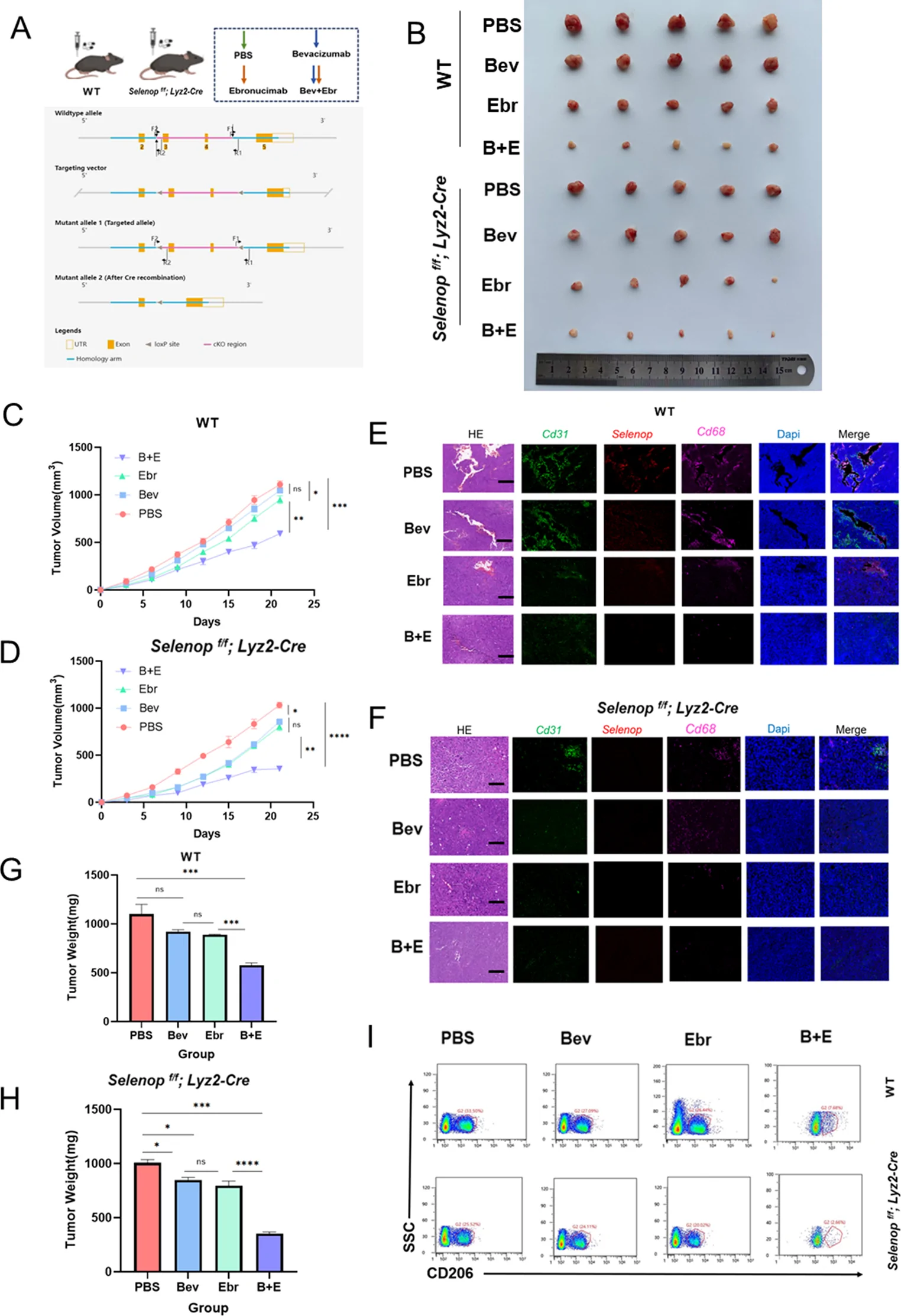
In vivo studies demonstrate that combination therapy with Bevacizumab and Ebronucimab effectively suppresses breast cancer angiogenesis in knockout mouse models. (**A**). Graphical abstract of the treatment regimen. (**B**). Photographs of resected mouse tumors. (**C**–**D**). Tumor growth curves of the two groups (* *P* < 0.05, ** *P* < 0.01, *** *P* < 0.001, ****P < 0.0001). (**E**–**F**). Multiplex immunofluorescence images of mouse tumors from different experimental groups to show the infiltration of Selenop+ macrophage and Cd31 to show the vascular invasion. Scale bar = 0.1 mm. (**G**–**H**). Comparison of mouse tumor endpoint weights between the two groups. Error bars show the mean ± SD. (* *P* < 0.05, ** *P* < 0.01, *** *P* < 0.001, ****P < 0.0001). (**I**). Flow cytometry analysis of mouse tumors from different experimental groups

To assess the in vivo relevance of this pathway, we used a myeloid-specific ***Selenop*** knockout model (***Selenop***^*f/f*;^ ***Lyz2***^Cre^) and implanted E0771 breast cancer cells (Fig. 8A). Knockout mice were assigned to PBS, bevacizumab, Ebronucimab, or combination treatment, with parallel wild-type groups analyzed.

Relative to wild-type controls, ***Selenop*** knockout mice demonstrated reduced tumor growth. Both bevacizumab and Ebronucimab monotherapy further decreased tumor volume, whereas the combination produced the strongest effect (Figs. 8B–D). Endpoint tumor weights followed the same pattern (Figs. 8G, H). Multiplex immunofluorescence revealed strong co-expression of ***Selenop*** and *Cd68* in wild-type tumors, whereas *Cd31* expression was lowest in tumors from knockout mice treated with the combination regimen (Figs. 8E, F). Flow cytometry demonstrated a lower proportion of M2 macrophages in knockout mice, with the greatest reduction observed in the combination group (Fig. 8I). These findings indicate that disruption of the SELENOP axis, particularly when combined with bevacizumab and Ebronucimab, attenuates tumor growth and vascular features associated with invasion in this model.

## Discussion

Breast cancer mortality is driven predominantly by metastatic progression rather than primary tumor burden, and VI represents a pivotal early event that enables tumor cells to enter the circulation [32]. Increasing evidence indicates that macrophage–endothelial communication shapes the angiogenic landscape and promotes invasive vascular phenotypes in breast cancer [33]. Previous studies have demonstrated that tumor-associated macrophages provide pro-angiogenic signals and modulate endothelial barrier properties, extracellular matrix remodeling, and vessel maturation, thereby facilitating tumor cell intravasation [34]. Building on these observations, our study identifies a distinct macrophage–endothelial metabolic program enriched in VI-positive breast tumors and provides functional evidence that this signaling axis promotes endothelial activation and VI.

A major finding of this study is the identification of SELENOP-expressing macrophages located in proximity to endothelial cells, as consistently demonstrated by single-cell RNA sequencing, spatial transcriptomics, and multiplex immunofluorescence. Although SELENOP has been studied primarily for its roles in systemic selenium transport and redox regulation, emerging evidence has linked this protein to immune cell adaptation, oxidative stress responses, chronic inflammation, and tumor-associated metabolic rewiring [35]. In cancer, SELENOP has been implicated in macrophage polarization, stromal remodeling, and tumor immune evasion [36, 37]. Our findings extend these observations by suggesting that SELENOP identifies a macrophage subset with enhanced pro-angiogenic potential in breast cancer and functions as a paracrine mediator that modulates endothelial metabolism and signaling.

Mechanistically, our findings identify endothelial LRP8 as a putative receptor mediating SELENOP uptake and downstream pathway activation. LRP8 belongs to the LDL receptor family, regulates lipid uptake, membrane homeostasis, and signal transduction in both neuronal and vascular systems [35]. Although its role in tumor angiogenesis remains incompletely explored, previous studies have suggested that LRP8 influences cholesterol trafficking, receptor localization, and cytoskeletal dynamics [38]. These processes are closely associated with endothelial migration and angiogenic sprouting. Our findings support a functional interaction between macrophage-derived SELENOP and endothelial LRP8, demonstrating that this ligand-receptor axis modulates cholesterol homeostasis, Hedgehog pathway activity, calcium signaling, and angiogenic behavior. Moreover, restoration of Hedgehog signaling through exogenous cholesterol supports the conclusion that sterol metabolism is a mechanistic component of SELENOP–LRP8–mediated endothelial activation.

The contribution of cholesterol metabolism to endothelial function has increasingly been recognized as an important determinant of angiogenesis and tumor vascularization. Cholesterol availability affects membrane raft composition, receptor clustering, and intracellular trafficking, thereby modulating endothelial responsiveness to angiogenic stimuli [38]. Dysregulated cholesterol pathways have been linked to abnormal tip/stalk cell dynamics, barrier breakdown, and pro-invasive vessel phenotypes [39, 40]. By demonstrating that SELENOP–LRP8 signaling alters endothelial cholesterol homeostasis, our findings integrate with this growing body of literature and indicate metabolically driven endothelial plasticity as a contributor to VI in breast cancer.

Our in vivo findings further support the biological relevance of this pathway. In the myeloid-specific *Selenop* knockout model, combined treatment with bevacizumab and Ebronucimab elicited greater suppression of tumor growth and vascular abnormalities compared with either monotherapy. Although anti-VEGF therapies demonstrated clinical benefit, their efficacy is frequently limited by metabolic compensation, endothelial adaptation, and recruitment of pro-angiogenic stromal components. Consequently, targeting cholesterol-related metabolic pathways may complement anti-angiogenic strategies. However, these observations are preclinical and should be interpreted with caution. Additional studies are imperative to evaluate treatment durability, potential resistance mechanisms, and safety considerations, including effects on lipid metabolism, immune function, and endothelial integrity. Validation across independent preclinical systems will be essential before advancing this therapeutic strategy toward clinical translation.

This study has several limitations. First, the patient cohort used for single-cell and spatial transcriptomic analyses was relatively small, potentially limiting the generalizability of cellular composition and signaling patterns. Second, macrophage–endothelial interactions were investigated using in vitro coculture systems that may not fully capture the complexity of in vivo cellular dynamics or biomechanical cues. Third, although the myeloid Selenop knockout model provides functional evidence supporting the proposed mechanism, murine tumors cannot fully reproduce the VI patterns observed in human breast cancer.

In summary, this study identifies a SELENOP^+^ macrophage–endothelial metabolic axis associated with VI in breast cancer. We propose a mechanistic model in which macrophage-derived SELENOP engages endothelial LRP8, thereby reprogramming cholesterol metabolism and promoting pro-angiogenic signaling. By integrating single-cell and spatial analyses with functional and in vivo assays, our findings advance the current understanding of macrophage-endothelial crosstalk within the TME and provide a conceptual framework for exploring metabolically targeted strategies to inhibit tumor VI. 

## Supplementary Information

Below is the link to the electronic supplementary material.

Supplementary Material 1 (DOCX 17.5 KB)

## Data Availability

The data that support the findings of this study are available from the corresponding author upon reasonable request.
