# Supplementary material for: SELENOP-LRP8-mediated macrophage-endothelial cell interaction promotes breast cancer vascular invasion by inducing metabolic reprogramming in endothelial cells
Source: Cell Mol Life Sci. 2026 Jul 31;83(1):399. doi: 10.1007/s00018-026-06359-y (PMC13713971; doi:10.1007/s00018-026-06359-y)
Supplement: Supplementary file 1 [file 18_2026_6359_MOESM1_ESM.docx]

**Supplementary Table 1. Baseline clinicopathological characteristics of breast cancer patients stratified by vascular tumor invasion status.**

| Patients ID | Age | Sex | Molecular subtype | TNM | Grade | Lymph nodes | Vascular invasion |
| --- | --- | --- | --- | --- | --- | --- | --- |
| 1 | 58 | female | Luminal B | T2N1M0 | 3 | Postive(1/15) | VI |
| 2 | 45 | female | TNBC | T2N1M0 | 3 | Postive(2/12) | VI |
| 3 | 62 | female | HER2 | T3N2M0 | 3 | Postive(5/16) | VI |
| 4 | 51 | female | TNBC | T2N1M0 | 2 | Postive(1/20) | VI |
| 5 | 70 | female | Luminal B | T2N0M0 | 3 | Postive(0/14) | VI |
| 6 | 65 | female | Luminal A | T1N0M0 | 1 | Negative(0/13) | NVI |
| 7 | 48 | female | Luminal B | T1N0M0 | 1 | Negative(0/12) | NVI |
| 8 | 55 | female | Luminal A | T2N0M0 | 2 | Negative(0/15) | NVI |
| 9 | 40 | female | HER2 | T2N0M0 | 2 | Negative(0/16) | NVI |
| 10 | 42 | female | TNBC | T1N0M0 | 2 | Negative(0/18) | NVI |
